# ﻿Five new species of the genus *Stigmus* Panzer (Hymenoptera, Crabronidae) from China, with a key to all Chinese species

**DOI:** 10.3897/zookeys.1204.123831

**Published:** 2024-06-10

**Authors:** Jinghong Li, Qiang Li, Li Ma

**Affiliations:** 1 Department of Entomology, College of Plant Protection, Yunnan Agricultural University, Kunming, Yunnan, 650201, China Yunnan Agricultural University Kunming China

**Keywords:** Digger wasps, identification key, new record, Pemphredoninae, taxonomy

## Abstract

Five new species of the genus *Stigmus* Panzer, 1804 are described and illustrated from Yunnan and Shaanxi provinces of China: *S.carinannulatus* Li & Ma, **sp. nov.**, *S.clypeglabratus* Li & Ma, **sp. nov.**, *S.flagellipilaris* Li & Ma, **sp. nov.**, *S.rugidensus* Li & Ma, **sp. nov.**, and *S.sulciconspicus* Li & Ma, **sp. nov.** In addition, *S.solskyi* Morawitz, 1864 is recorded in China for the first time. An illustrated key to known and new species of the genus *Stigmus* Panzer from China is provided.

## ﻿Introduction

The genus *Stigmus* Panzer was erected by Panzer with *Stigmuspendulus* Panzer, 1804 as its type species. The classification system used in this study follows Melo (1999). *Stigmus* belongs to the Hymenoptera: Crabronidae: Pemphredoninae: Pemphredonini: Stigmina. All members of this genus are predatory towards aphids that are found on woody plants except that some species of *Rhopalosiphum* and *Aphis* that occur on herbaceous plants (Tsuneki 1954; Krombein 1958). The overwhelming majority of species within *Stigmus* nest in wood in some form. Reported nesting sites include in twigs, dead trees, or structural lumber of abandoned borings by other insects, principally beetle larvae, in borings made by themselves in the pith of twigs or stems, and in abandoned galls of other insects (Smith 1923; Tsuneki 1970; Krombein 1973). The nests usually consist of a linear series of cells separated by partitions of small pieces of pith of the wood substrate (Krombein 1955).

The main diagnostic characteristics of the subtribe Stigmina include forewing with two or fewer discoidal cells and one recurrent vein and a large stigma; forewing with elongate marginal cell, closed apically, larger than stigma; in dorsal view, petiole length significantly longer than its width. *Stigmus* can be easily distinguished from the similar genus *Carinostigmus* based on the following identifying characteristics: vertex with micropore field; hindwing submedian cell normal size; occipital carina usually incomplete, not ending to midventral line, suddenly ending at posterior ridge of stomal hollow; interantennal tubercle degenerative; petiole with carinae. *Carinostigmus* has the following characters: vertex without micropore field; hindwing submedian cell degenerative; occipital carina complete, ending to midventral line; interantennal tubercle distinct; petiole usually smooth (Krombein 1973, 1984; Bohart and Menke 1976; Budrys 1987; Finnamore 1995).

The genus currently consists of 30 species and four subspecies worldwide, distributed across four major zoogeographic regions, in which the majority of species occurred in the Nearctic region (10 species and 2 subspecies) and the Palearctic region (10 species); additionally, six species and two subspecies occurred in the Oriental region, while the Neotropical region has a relatively low distribution with only two species. One species occurs in the Palearctic and Oriental regions, and one species in the Nearctic and Neotropical regions. Currently, eight species and two subspecies have been recorded in China, among them, four species and two subspecies are distributed in the Oriental region, while three species are distributed in the Palearctic; additionally, there is one species that is found in both the Palearctic and Oriental regions of China (Morawitz 1864; Packard 1867; Kohl 1892; Rohwer 1911; Tsuneki 1954, 1971; Krombein 1973; Kolesnikov 1977; Allen 1987; Budrys 1987, 1995; Uffen 1997, 1998; Jones 2001; Amarante 2002; Terayama 2012; Ratzlaff 2016; Mokrousov 2017; Pulawski 2024).

In the current study, five new species of *Stigmus* from China are described and illustrated as *S.carinannulatus* Li & Ma, sp. nov., *S.clypeglabratus* Li & Ma, sp. nov., *S.flagellipilaris* Li & Ma, sp. nov., *S.rugidensus* Li & Ma, sp. nov., and *S.sulciconspicus* Li & Ma, sp. nov., and one newly recorded species from Yunnan Province of China is reported. Additionally, an illustrated identification key to the Chinese *Stigmus* is provided.

## ﻿Materials and methods

The specimens examined in this study are deposited in the Insect Collections of Yunnan Agricultural University, Kunming, Yunnan, China (YNAU). Specimens were observed with the help of an Olympus stereomicroscope (SZ Series) with an ocular micrometer. The photographs were taken with VHX-5000 and edited by using Adobe Photoshop 8.0. For the terminology we mainly followed Tsuneki (1954), Bohart and Menke (1976) and Bashir et al. (2019). The abbreviations and definitions utilized in the text are as follows:

**opaque area** small area located between the ocellar triangle area and eye, close to the eye;

**triangular area** area enclosed by scrobal suture, omaulus and hypersternaulus;

**BL** body length;

**HLD** head length in dorsal view, the distance from frons to occipital margin, medially;

**HLF** head length in frontal view, the distance from vertex to clypeal margin, medially;

**HW** head width in dorsal view, maximum;

**EW** eye width in lateral view, maximum;

**Ewd** eye width in frontal view, maximum;

**TW** gena width in lateral view, maximum;

**EL** eye length in lateral view, maximum;

**POD** postocellar distance, distance between inner margins of hind ocelli, dorsally;

**OOD** ocellocular distance, distance between outer margin of hind ocellus and nearest inner orbit, dorsally;

**OCD** ocello-occipital distance, distance between posterior margin of hind ocellus and occipital margin, dorsally;

**PW** petiole width, maximum, dorsally;

**PL** petiole length, dorsally;

**WTI** width of metasomal tergum I, maximum, dorsally;

**LTI** length of gastral tergum I, maximum, dorsally;

**HFL** length of hind femur, maximum;

**HTL** length of hind tibia, maximum.

## ﻿Taxonomy

### ﻿Genus Stigmus

Taxon classificationAnimaliaHymenopteraCrabronidae

Panzer, 1804

CAEDF294-88B1-5ED1-966D-6F0FE8D29122

#### Type species.

*Stigmuspendulus* Panzer, 1804.

##### ﻿Key to the species of *Stigmus* from China, including males and females

Females of *S.flagellipilaris* Li & Ma, sp. nov., *S.capoblongus* Bashir & Ma and males of *S.fronticoncavus* Bashir & Ma, *S.sulciconspicus* Li & Ma, sp. nov., and *S.interruptus* Bashir & Ma are unknown. PR and OR represent Palearctic and Oriental Regions, respectively.

**Table:** 

1	Clypeus deeply impressed, not produced (OR)	***S.fronticoncavus* Bashir & Ma**
–	Clypeus flat or slightly convex, slightly (Fig. 1A) or strongly produced	**2**
2	Triangular area with sturdy reticulation (Fig. 6D) or striations	**3**
–	Triangular area smooth and shiny (Fig. 1E)	**4**
3	Scutellum coriaceous, without longitudinal impressed line medially (Fig. 6C); mesopleuron episcrobal area and triangular area with sturdy reticulation (Fig. 6D); in male, clypeus with dense, silvery, short setae, free margin of clypeus strongly produced and nearly truncate medially (Fig. 6H) (PR)	***S.solskyi* Morawitz**
–	Scutellum shiny, with single, slender longitudinal line medially; mesopleuron episcrobal area with dense, longitudinal striations, and triangular area with distinct striations anteriorly, smooth and shiny posteriorly; in male, clypeus without setae, free margin of clypeus slightly produced and with two triangular teeth medially (OR)	***S.shirozuialishanus* Tsuneki**
4	Ventral surface of petiole shiny, without rugae (OR)	***S.kansitakuanus* Tsuneki**
–	Ventral surface of petiole with a few strong, longitudinal rugae medially and posteriorly	**5**
5	Scrobal suture inconspicuous, lacking (Fig. 1E) or only with single weak rugae (Fig. 3E), not crenate	**6**
–	Scrobal suture narrow or broad, weakly or distinctly crenate (Fig. 4D)	**9**
6	Hindwing media diverging before cu-a (Fig. 7A) (OR)	**7**
–	Hindwing media diverging beyond cu-a	**8**
7	Occipital carina complete, ending to midventral line (Fig. 1C); scutum with conspicuous, longitudinal striations, posterior area with dense, slender, short, longitudinal rugae (Fig. 1D); posterior area of mesopleuron with sparse, short longitudinal rugae, and episcrobal area smooth, without striations (Fig. 1E)	***S.carinannulatus* Li & Ma, sp. nov.**
–	Occipital carina incomplete, not ending to midventral line, suddenly ended at posterior ridge of stomal hollow (Fig. 3B); scutum without striations or rugae (Fig. 3D); posterior area of mesopleuron smooth, without rugae, and episcrobal area with dense, slender, longitudinal striations (Fig. 3E)	***S.flagellipilaris* Li & Ma, sp. nov.**
8	Opaque area larger than hind ocellus (Fig. 5B); pronotal collar with complete, transverse carina anteriorly (Fig. 5C); dorsal surface of petiole with sturdy, irregular rugae anteriorly and medially, and several sturdy, longitudinal rugae posteriorly (Fig. 5F) (OR)	***S.sulciconspicus* Li & Ma, sp. nov.**
–	Opaque area smaller than hind ocellus; pronotal collar with incomplete, transverse carina anteriorly, narrowly emarginated in middle; dorsal surface of petiole with two strong longitudinal carinae, and irregular, strong rugae anteriorly and medially (PR)	***S.denticorneus* Bashir & Ma**
9	Hindwing media diverging before cu-a (Fig. 7A) (OR)	**10**
–	Hindwing media diverging beyond cu-a	**13**
10	Pronotal collar with complete lateral rugae; lateral surface of propodeum with dense, sturdy or slender, oblique, longitudinal rugae anteriorly and medially	**11**
–	Pronotal collar with incomplete lateral rugae, only distinct in posterior area (Fig. 3C); lateral surface of propodeum smooth, without rugae anteriorly and medially (Fig. 3D)	**12**
11	Ventral gena shiny, with dense, large punctures mixed with several irregular rugae laterally; inner orbital furrow broadened, with slender rugae; scutum shiny, with sparse, midsize to large punctures	***S.lobomelanicus* Bashir & Ma**
–	Ventral gena smooth, impunctate and without rugae; inner orbital furrow lacking; scutum moderately matt, with sparse, tiny punctures, posterior area with several sturdy, short, longitudinal rugae	***S.murotai* (Tsuneki)**
12	Vertex with sparse, large punctures (Fig. 4B); occipital carina narrow, coarsely crenulate dorsally, and somewhat broadened, distinctly crenulate ventrally; pronotal lobe black (Fig. 4D); scutum shiny, with sparse, large punctures (Fig. 4C); in female, pygidial area moderately matt, basal area with several midsize punctures (Fig. 4G)	***S.rugidensus* Li & Ma, sp. nov.**
–	Vertex with sparse, fine punctures (Fig. 2B); occipital carina much narrowed, not crenulate; pronotal lobe yellowish (Fig. 2D); scutum moderately matt, with sparse, tiny punctures (Fig. 2C); in female, pygidial area shiny, without punctures (Fig. 2G)	***S.clypeglabratus* Li & Ma, sp. nov.**
13	Lateral surface of propodeum smooth, without rugae anteriorly and medially; posterior area of mesopleuron with sparse, short, longitudinal rugae (PR)	***S.capoblongus* Bashir & Ma**
–	Lateral surface of propodeum with dense, slender or sturdy, oblique, longitudinal rugae; posterior area of mesopleuron smooth, without rugae (OR)	**14**
14	Vertex with several midsize punctures; anterior area of pronotal collar with incomplete, transverse carina, narrowly emarginated in middle; scutum with sparse, large punctures, anterior and posterior areas with dense, longitudinal striations	***S.interruptus* Bashir & Ma**
–	Vertex without puncture; anterior area of pronotal collar with complete, transverse carina; scutum with sparse, tiny punctures, without striations	**15**
15	Hypersternaulus narrowed, not crenate; posterior surface of propodeum with shallow and somewhat narrow median groove; PL/PW ~ 5; in female, pygidial area impunctate, with dense, weak, longitudinal striations	***S.convergensami* Tsuneki**
–	Hypersternaulus broadened, distinctly crenate; posterior surface of propodeum without conspicuous groove; PL/PW ~ 3; in female, pygidial area with two lines of large punctures medially, without striations	***S.japonicus* Tsuneki**

##### ﻿Species accounts

### ﻿ Stigmus carinannulatus

Taxon classificationAnimaliaHymenopteraCrabronidae

Li & Ma sp. nov.

723F74A0-522E-5F07-995E-0BB05C3FF88A

https://zoobank.org/CBECB838-47C0-4C4F-AA4B-ED9E9681ECDC

Figs 1A–N , 7A, B

#### Type material.

***Holotype***: China • ♀; Yunnan, Tengchong City; 25°1′N, 92°28′E; 11.VIII.2011; coll. Jujian Chen; sweep net (YNAU). ***Paratypes***: China • 20♂♂; Yunnan, Kunming City, Yunnan Agricultural University; 25°7′N, 102°44′E; 12.IV.2023 (5♂♂), 12.VI.2023 (9♂♂), 19.VIII.2023 (6♂♂); 1910 m elev.; coll. Jinghong Li; sweep net (YNAU); China • 1♂; Yunnan, Baoshan City, Longyang District, Lujiang Country, Pumanshao Village; 24°56′N, 98°47′E; 21.VII.2006; 1951 m elev.; coll. Zhongshi Zhou; sweep net (YNAU).

#### Diagnosis.

The new species can be easily separated from the similar species *S.denticorneus* Bashir & Ma, 2019 by the following characters: hindwing media diverging before cu-a; occipital carina complete, ending to midventral line; anterior area of pronotal collar with complete, transverse carina; scutum with several, large punctures, anterior area with distinct, longitudinal striations, posterior area with dense, fine, short, longitudinal rugae; posterior area of mesopleuron with sparse, short, longitudinal rugae, episcrobal area smooth, without striation. *Stigmusdenticorneus* has the following characters: hindwing media diverging beyond cu-a; occipital carina incomplete, not ending to midventral line, suddenly ended at posterior ridge of stomal hollow; anterior area of pronotal collar with incomplete, transverse carina, narrowly emarginated in middle; anterior area of scutum with dense, large punctures, remainder with sparse, midsize to large punctures; posterior area of mesopleuron smooth, without rugae, episcrobal area with dense, longitudinal striations.

#### Description.

**Female. *Measurements.*** ♀, BL: 4.6 mm; HW: HLD: HLF = 78: 54: 59; HW: EWd: EW: TW: EL = 78: 23: 22: 28: 51; POD: OOD: OCD = 8: 14: 15; length of scape: length of pedicel: length of flagellomere I: width of flagellomere I: length of flagellomere II: width of flagellomere II = 21: 9: 8: 5: 8: 5; PL: PW: LTI: WTI: HFL: HTL = 36: 11: 40: 46: 48: 55. ♂, BL: 3.5–4.6 mm; HW: HLD: HLF = 53: 31: 42; HW: EWd: EW: TW: EL = 53: 15: 20: 12: 36; POD: OOD: OCD = 5.5: 10: 11; length of scape: length of pedicel: length of flagellomere I: width of flagellomere I: length of flagellomere II: width of flagellomere II = 13: 5: 5: 3: 5: 3; PL: PW: LTI: WTI: HFL: HTL = 24: 5: 25: 26: 33: 57.

***Color pattern.*** Body black; clypeus with reddish brown to dark brown band subapically; mandible fulvous except reddish brown apically; labrum dark brown; palpi, scape, pedicel, tegula and pronotal lobe fulvous; flagellomeres I–V beneath fulvous, above brown, remainder dark brown; forewing veins brown; fore and mid legs: fulvous except coxa largely and femur medially dark brown; hind leg: trochanter, basal 1/4 of tibia and tarsus fulvous, remainder dark brown; gastral sterna IV-VI fulvous to dark brown; setae on clypeus and mandible sparse and golden.

**Figure 1.**
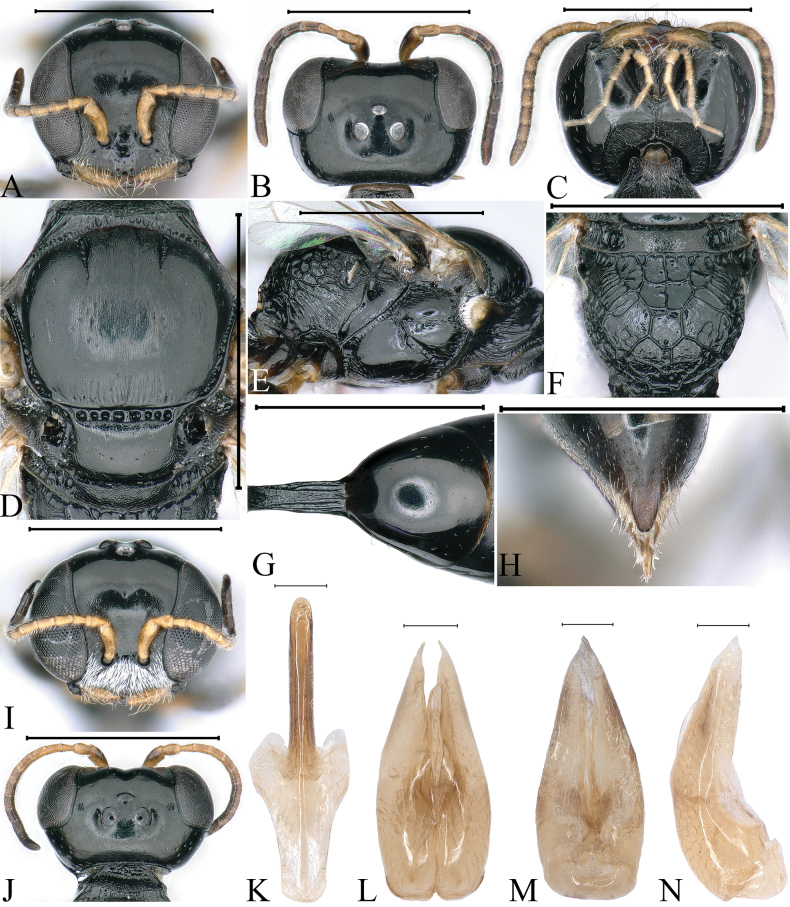
*Stigmuscarinannulatus* Li & Ma, sp. nov. (**A–H** female **I–N** male) **A, I** head, frontal view **B, J** head, dorsal view **C** head, ventral view **D** collar, scutum, scutellum and metanotum, dorsal view **E** thorax, lateral view **F** propodeum, dorsal view **G** petiole, dorsal view **H** pygidial plate, dorsal view **K** gastral tergum VIII, ventral view **L** male genitalia, dorsal view **M** male genitalia, ventral view **N** male genitalia, lateral view. Scale bars: 1 mm (**A–J**); 0.1 mm (**K–N**).

***Head.*** Mandible tridentate apically, median tooth large (Fig. 1A). Labrum with two distinct cornuted teeth apically. Clypeus shiny, nearly flat, with sparse, midsize punctures; free margin of clypeus slightly produced and with two distinct triangular teeth medially, slightly reflected, area between two teeth deeply emarginated (Fig. 1A). Scapal hollow moderately matt, coriaceous, somewhat shallow and defined, provided with one vestigial minute tubercle medially, not spined (Fig. 1A). Frontal furrow very fine and weakly impressed, inconspicuously (Fig. 1A). Median and upper frons shiny, with sparse, fine punctures, gently convex (Fig. 1A). Ocellar triangle area flat, shiny, impunctate, area near eyes with dense, short, impressed lines, opaque area smaller than hind ocellus (Fig. 1B). Vertex shiny, impunctate (Fig. 1B). Gena shiny, smooth and impunctate (Fig. 1C). Head from above with temples rarely convergent posteriorly, subquadrate (Fig. 1B). Occipital carina complete, ending to midventral line, dorsal area much narrowed, not crenulate, ventral area gently broadened, coarsely crenate (Fig. 1C); inner and outer orbital furrows lacking (Fig. 1A).

***Mesosoma.*** Pronotal collar with strong, transverse carina anteriorly, and with incomplete lateral rugae, only distinct in posterior area, without antero-lateral corner (Fig. 1D). Scutum moderately matt, with several large punctures, anterior area with dense, conspicuous, longitudinal striations, posterior area with dense, fine, short, longitudinal rugae (Fig. 1D); admedian line weakly impressed, extending to 1/2 of scutum length; notaulus deeply grooved and crenulate, reaching 2/5 of scutum length; parapsidal line distinct (Fig. 1D). Scutellum shiny, with sparse, fine punctures, without medial longitudinal line (Fig. 1D). Metanotum weakly coriaceous (Fig. 1D). Mesopleuron shiny, posterior area with several slender, short, longitudinal rugae, episcrobal area shiny and smooth (Fig. 1E); omaulus and hypersternaulus broadened, distinctly crenate, scrobal suture complete and inconspicuous, just with single longitudinal rugae (Fig. 1E). Propodeal enclosure triangular medially, with three sturdy longitudinal median rugae, and several transvers rugae, with sparse sturdy, oblique, longitudinal rugae laterally (Fig. 1F); posterior surface of propodeum with sparse irregular rugae, without conspicuous median groove (Fig. 1F); lateral surface of propodeum with dense, oblique, longitudinal rugae anteriorly and medially, and irregular reticulation posteriorly (Fig. 1E).

***Legs.*** Outer surface of hind tibia with three long, slender, fulvous to dark brown spines.

***Wings.*** Forewing venation typical for genus *Stigmus*, hindwing media diverging before cu-a.

***Metasoma.*** Dorsal surface of petiole subquadrate, moderately convex and widened toward apex slightly, and with two sturdy, longitudinal, median carinae, area between carinae with dense, fine, irregular rugae, median and posterior areas with two sturdy, longitudinal, lateral rugae on each side (Fig. 1G). Lateral surface of petiole with three strong, longitudinal rugae medially and posteriorly (Fig. 7A). Ventral surface of petiole with a few strong, short, longitudinal rugae posteriorly. Gastral terga shiny, impunctate, gastral sternum VI with sparse, fine punctures apically (Fig. 1H). Pygidial area moderately matt, broadly triangular, apex truncate, with longitudinal micro-striations (Fig. 1H).

**Male.** Same as female except tegula dark brown; setae on clypeus dense, silvery, short (Fig. 1I); mandible bidentate apically (Fig. 1I); free margin of clypeus slightly produced, nearly truncate medially, and with shallow emargination (Fig. 1I); head from above with temples somewhat roundly convergent posteriorly; dorsal area of occipital carina much narrowed, coarsely crenulate, and ventral area somewhat broadened, distinctly crenate (Fig. 1J); flagellomeres without tyloids, setae normal; gastral sterna impunctate (Fig. 1J).

#### Distribution.

China (Yunnan).

#### Etymology.

The name, *carinannulatus*, is derived from the Latin *carin*- (= carina) and the Latin word *annulatus* (= annular), referring to the complete occipital carina.

### ﻿ Stigmus clypeglabratus

Taxon classificationAnimaliaHymenopteraCrabronidae

Li & Ma sp. nov.

7EA16859-7141-5352-B447-6F9EF3FE767A

https://zoobank.org/A5CB1E7E-541A-4D20-ABB4-E62DBDE9ABA6

Figs 2A–M , 7C, D

#### Type material.

***Holotype***: China • ♀; Shaanxi, Hanzhong City, Liuba County, Zibai Mountain; 33°40′N, 106°43′E; 3.VIII.2004; 1632 m elev.; coll. Min Shi; sweep net (YNAU). ***Paratypes***: 3♂♂, same data as for holotype, except coll. Min Shi, Qiong Wu (YNAU).

#### Diagnosis.

Differs from *S.japonicus* Tsuneki, 1954 by hindwing media diverging before cu-a; lateral surface of propodeum smooth and shiny anteriorly and medially; pronotal collar with incomplete lateral rugae, just distinct in posterior area; gena impunctate dorsally; opaque area larger than hind ocellus; in male, clypeus smooth and impunctate, and with several setae on free margin, yellowish and short; in female, pygidial area smooth, impunctate. *Stigmusjaponicus* has the following characters: hindwing media diverging beyond cu-a; lateral surface of propodeum with dense, slender or sturdy, oblique, longitudinal rugae anteriorly and medially; pronotal collar without lateral rugae; gena with sparse, midsize punctures dorsally; opaque area smaller than hind ocellus; in male, clypeus with dense, tiny punctures, setae on clypeus dense, silvery, and short; in female, pygidial area with two lines of large punctures medially.

#### Description.

**Female. *Measurements.*** ♀, BL: 4.2 mm; HW: HLD: HLF = 61: 36: 47; HW: EWd: EW: TW: EL = 61: 16: 21: 16: 39; POD: OOD: OCD = 5: 11: 16; length of scape: length of pedicel: length of flagellomere I: width of flagellomere I: length of flagellomere II: width of flagellomere II = 18: 6: 6: 3: 7: 3.5; PL: PW: LTI: WTI: HFL: HTL = 26: 8: 32: 35: 35: 40. ♂, BL: 3–3.8 mm; HW: HLD: HLF = 63: 32: 48; HW: EWd: EW: TW: EL = 63: 19: 23: 13: 41; POD: OOD: OCD = 6.5: 11: 14; length of scape: length of pedicel: length of flagellomere I: width of flagellomere I: length of flagellomere II: width of flagellomere II = 16: 7: 5: 3: 6: 3; PL: PW: LTI: WTI: HFL: HTL = 26: 8: 29: 34: 34: 40.

***Color pattern.*** Body black; clypeus with reddish brown band subapically; mandible yellowish except reddish brown apically; labrum and dorsal scape fulvous; palpi and ventral scape ivory; pedicel, pronotal lobe, tegula and forewing veins yellowish; flagellomeres beneath and I-II above fulvous, remainder reddish brown; fore and mid legs: yellowish to fulvous except outer margin of femur somewhat brown, coxa dark brown largely; hind leg: coxa apically, trochanter, basal 1/2 of tibia, tarsi yellowish to fulvous, remainder dark brown; gaster dark brown, gastral sterna IV–VI bright yellow largely; setae on clypeal margin and mandible sparse, golden and long.

**Figure 2.**
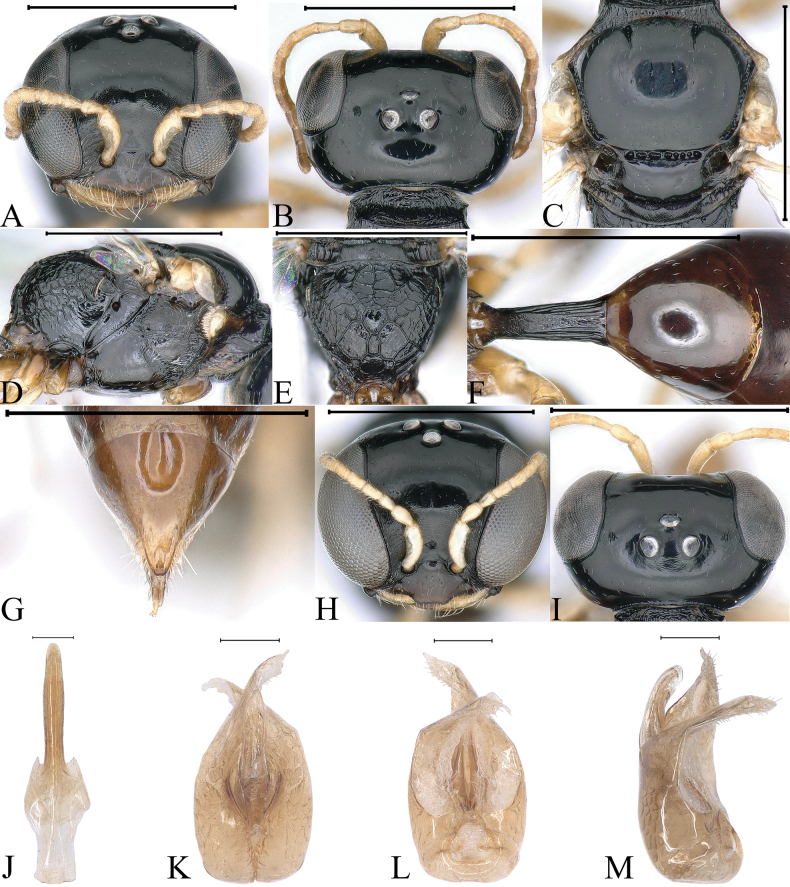
*Stigmusclypeglabratus* Li & Ma, sp. nov. (**A–G** female **H–M** male) **A, H** head, frontal view **B, I** head, dorsal view **C** collar, scutum, scutellum and metanotum, dorsal view **D** thorax, lateral view **E** propodeum, dorsal view **F** petiole, dorsal view **G** pygidial plate, dorsal view **J** gastral tergum VIII, ventral view **K** male genitalia, dorsal view **L** male genitalia, ventral view **M** male genitalia, lateral view. Scale bars: 1 mm (**A–I**); 0.1 mm (**J–M**).

***Head.*** Mandible tridentate apically, median tooth large. Labrum with two distinct triangular teeth apically (Fig. 2A). Clypeus smooth, shiny, flat; free margin of clypeus slightly produced and with two triangular teeth medially, slightly reflected, area between two teeth with shallow emargination (Fig. 2A). Scapal hollow shiny, shallow, and broad, not clearly defined, provided with one vestigial minute tubercle medially; frontal furrow vestigial; median and upper frons shiny, with sparse, tiny punctures (Fig. 2A). Ocellar triangle area flat, shiny, impunctate, area near eyes with dense, short, impressed lines, opaque area smaller than hind ocellus (Fig. 2B). Vertex shiny, with sparse, tiny punctures (Fig. 2B). Gena shiny, impunctate. Head from above with temples rarely convergent posteriorly, subquadrate (Fig. 2B). Occipital carina incomplete, not ending to midventral line, suddenly ended at posterior ridge of stomal hollow, not dentate, occipital carina much narrowed, not crenulate. Inner and outer orbital furrows lacking (Fig. 2A).

***Mesosoma.*** Pronotal collar with strong, transverse carina anteriorly, and with incomplete lateral rugae, only distinct in posterior area, without antero-lateral corner (Fig. 2C). Scutum moderately matt, with sparse, tiny punctures; admedian line distinctly impressed, extending to 2/5 scutum length; notaulus deeply grooved and crenulate, reaching quarter of scutum length; parapsidal line weakly impressed (Fig. 2C). Scutellum shiny, with sparse, fine punctures, and median longitudinal line weakly impressed (Fig. 2C). Metanotum weakly coriaceous (Fig. 2C). Mesopleuron smooth and shiny, posterior area smooth without rugae, episcrobal area with dense, slender, longitudinal rugae posteriorly; scrobal suture, omaulus and hypersternaulus broadened, slightly crenate, scrobal suture complete (Fig. 2D). Propodeal enclosure triangular medially, and with three sturdy, longitudinal rugae and sparse, irregular, transvers rugae, lateral area with irregular, short rugae; posterior surface of propodeum with sturdy reticulation, and without conspicuous median groove (Fig. 2E); lateral surface of propodeum smooth and shiny anteriorly and medially, and with irregular reticulation posteriorly (Fig. 2D).

***Legs.*** Outer surface of hind tibia with three long, slender, fulvous to dark brown spines.

***Wings.*** Forewing venation typical for genus *Stigmus*, hindwing media diverging before cu-a.

***Metasoma.*** Dorsal surface of petiole subquadrate, gently convex and widened toward apex slightly, and with two sturdy oblique, longitudinal carinae forming V-shaped medially, area between V-shaped carinae with irregular rugae, medial and posterior areas with a few sturdy, longitudinal rugae on each side (Fig. 2F); lateral surface of petiole with several strong, longitudinal rugae medially and posteriorly (Fig. 7C); ventral surface of petiole with four strong, short, longitudinal rugae posteriorly. Gastral terga shiny, impunctate, gastral sternum VI moderately matt, and with dense fine punctures (Fig. 2G). Pygidial area smooth and shiny, broadly triangular (Fig. 2G).

**Male.** Almost same as female except mandible fulvous basally and medially; fore and mid legs yellowish to fulvous; hind femur dark brown largely, remainder yellowish; mandible bidentate apically; clypeus moderately convex, clypeal margin broadly produced, and nearly truncate medially (Fig. 2H); head from above with temples gradually convergent posteriorly (Fig. 2I); occipital carina narrowed, distinctly crenulate dorsally, and ventral area somewhat broadened, coarsely crenate (Fig. 2I); flagellomeres without tyloids, setae normal (Fig. 2I).

#### Distribution.

China (Shaanxi).

#### Etymology.

The name, *clypeglabratus*, is derived from the Latin *clype*- (= clypeus) and the Latin word *glabratus* (= smooth), referring to the smooth and impunctate clypeus.

### ﻿ Stigmus flagellipilaris

Taxon classificationAnimaliaHymenopteraCrabronidae

Li & Ma sp. nov.

9F815973-B253-587B-BACC-15AC43A94412

https://zoobank.org/9DD287CF-D292-49D8-88E2-2FE6D9BB599B

Figs 3A–K , 8A

#### Type material.

***Holotype***: China • ♂; Yunnan, Tengchong city, Shabadi Village; 25°23'N, 98°42'E; 2–18.IV.2020; 1739 m elev.; coll. Lang Yi; Malaise trap (YNAU). ***Paratypes***: China • 1♂; Yunnan, Baoshan city, Longyang District, Lujiang Country, Pumanshao Village; 24°56′N, 98°47′E; 21.VII.2006; 1951 m elev.; coll. Zhongshi Zhou; sweep net (YNAU); China • 1♂; Yunnan, Wenshan City, Maguan County, Wazishan Village; 22°51′N, 104°23′E; 13.VIII.2017; 1722 m elev.; coll. Li Ma; sweep net (YNAU).

#### Diagnosis.

Differs from *S.japonicus* by hindwing media diverging before cu-a; scrobal suture inconspicuous, just single weak rugae; opaque area smaller than hind ocellus; median and upper frons with several large punctures; vertex shiny, and with sparse, fine punctures. *Stigmusjaponicus* has the following characters: hindwing media diverging beyond cu-a; scrobal suture broadened, distinctly crenate; opaque area larger than hind ocellus; medial and upper frons with sparse, fine punctures; vertex moderately matt, with sparse, midsize punctures.

#### Description.

**Male. *Measurements.*** ♂, BL: 3.2–4.3 mm; HW: HLD: HLF = 69: 44: 54; HW: EWd: EW: TW: EL = 69: 20: 19: 23: 43; POD: OOD: OCD = 8: 12: 12; length of scape: length of pedicel: length of flagellomere I: width of flagellomere I: length of flagellomere II: width of flagellomere II = 16: 6: 7: 4: 7: 4; PL: PW: LTI: WTI: HFL: HTL = 33: 7: 34: 32: 39: 50.

***Color pattern.*** Body black; mandible yellowish except reddish brown apically; labrum, scape, pedicel and pronotal lobe fulvous; palpi yellowish; flagellomeres reddish brown except I–IV beneath fulvous; tegula brown; forewing veins fulvous to brown; fore and mid legs: trochanter, base and apex of femur, tibia largely, tarsi yellowish, remainder dark brown; hind leg: coxa apically, trochanter, base and apex of femur, tibia largely, tarsi yellowish, remainder dark brown; setae on clypeus and lateral upper frons silvery; mandible with sparse golden setae.

**Figure 3.**
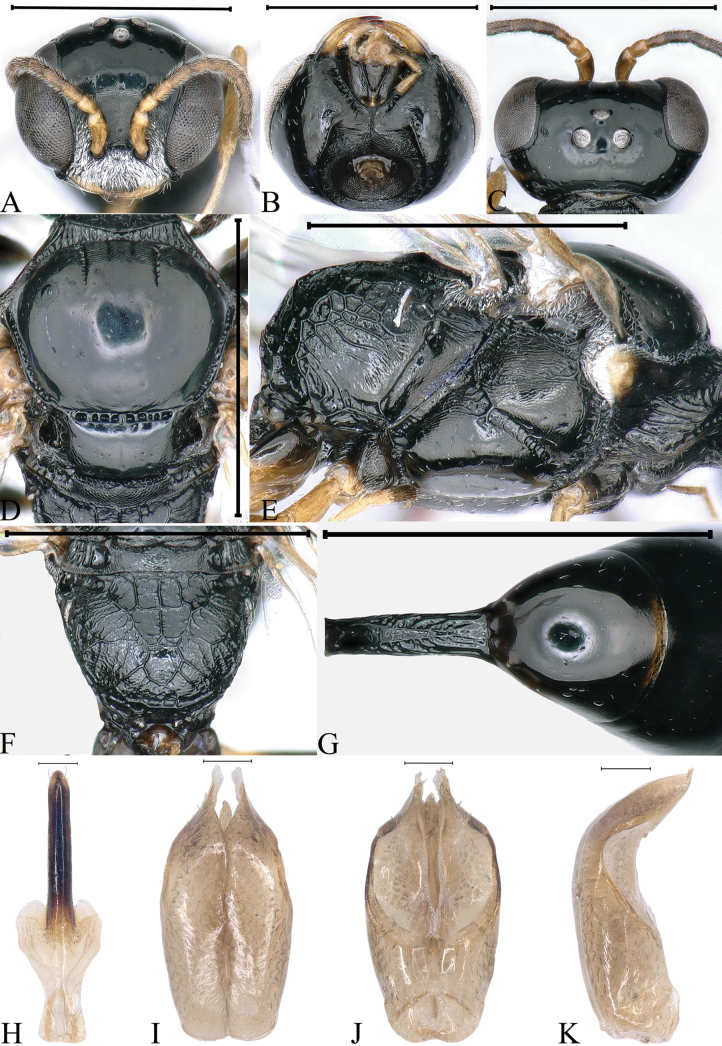
*Stigmusflagellipilaris* Li & Ma, sp. nov. (male) **A** head, frontal view **B** head, ventral view **C** head, dorsal view **D** collar, scutum, scutellum and metanotum, dorsal view **E** thorax, lateral view **F** propodeum, dorsal view **G** petiole, dorsal view **H** gastral tergum VIII, ventral view **I** male genitalia, dorsal view **J** male genitalia, ventral view **K** male genitalia, lateral view. Scale bars: 1 mm (**A–G**); 0.1 mm (**H–K**).

***Head.*** Mandible bidentate apically (Fig. 3A). Labrum subquadrate (Fig. 3A). Clypeus nearly flat, with dense, tiny punctures; free margin of clypeus slightly produced and nearly truncate medially, with shallow emargination (Fig. 3A). Scapal hollow matt, distinctly coriaceous, somewhat shallow, provided with one vestigial minute tubercle medially; frontal furrow weakly impressed; medial and upper frons shiny, with several large punctures, slightly convex (Fig. 3A). Ocellar triangle area flat, shiny, impunctate, area near eyes with dense, short, impressed lines, opaque area smaller than hind ocellus (Fig. 3C). Vertex shiny, with sparse, fine punctures (Fig. 3C). Gena shiny, with sparse, midsize to large punctures dorsally; ventral gena shiny, smooth, impunctate (Fig. 3B). Head from above with temples gradually convergent posteriorly (Fig. 3C). Occipital carina incomplete, not ending to midventral line, suddenly ended at posterior ridge of stomal hollow, dorsal area much narrowed, not crenulate, ventral area gently broadened, distinctly crenate (Fig. 3B). Inner and outer orbital furrows lacking (Fig. 3A, B). Flagellomeres without tyloids, with longish and dense setae (Fig. 3A).

***Mesosoma.*** Pronotal collar with strong, transverse carina anteriorly, and with incomplete lateral rugae, only distinct in posterior area, without antero-lateral corner (Fig. 3D). Scutum moderately matt, with several inconspicuous, large punctures; admedian line distinctly impressed, extending to 2/5 scutum length; notaulus deeply grooved and crenulate, reaching 1/3 of scutum length; parapsidal line distinctly impressed (Fig. 3D). Scutellum matt, with sparse, midsize punctures, and median, longitudinal line weakly impressed (Fig. 3D). Metanotum distinctly coriaceous (Fig. 3D). Mesopleuron moderately matt, with fine sculptures and several large punctures, posterior area smooth, without rugae, episcrobal area with dense, slender, longitudinal striations; omaulus and hypersternaulus broadened, distinctly crenate, scrobal suture inconspicuous, just with several single rugae (Fig. 3E). Propodeal enclosure triangular medially, with three sturdy, longitudinal rugae and sparse, strong, transvers rugae, lateral area with irregular rugae mixed with a few, sturdy, oblique, longitudinal rugae; posterior surface of propodeum without distinct median groove, with sparse, strong, transverse rugae, remainder with sturdy reticulation (Fig. 3F); lateral surface of propodeum with contiguous, slender or sturdy, oblique, longitudinal rugae anteriorly and medially, and irregular reticulation posteriorly (Fig. 3E).

***Legs.*** Outer surface of hind tibia with three long, slender, fulvous to dark brown spines.

***Wings.*** Forewing venation typical for genus *Stigmus*, hindwing media diverging before cu-a.

***Metasoma.*** Dorsal surface of petiole subquadrate, moderately convex and widened toward apex slightly, and with two sturdy oblique, longitudinal carinae forming V-shaped medially, area between carina gently convex, and with a few sturdy, oblique, longitudinal rugae on each side (Fig. 3G); lateral surface of petiole with several strong, longitudinal rugae (Fig. 8A); ventral surface of petiole with a few sturdy, short, longitudinal rugae posteriorly.

**Female.** Unknown.

#### Distribution.

China (Yunnan).

#### Etymology.

The specific name, *flagelli*, is derived from the Latin *flagell*- (= flagellum) and the Latin word *pilaris* (= crinal), referring to the flagella without tyloids, and with long setae and dense pilosity.

### ﻿ Stigmus rugidensus

Taxon classificationAnimaliaHymenopteraCrabronidae

Li & Ma sp. nov.

D828EB4A-2DDA-5CDC-93B7-48F475F1F7EA

https://zoobank.org/B9DD920D-D1B6-46C6-A777-64CF2F1FB196

Figs 4A–M , 7E, F

#### Type material.

***Holotype***: China • ♀; Yunnan, Kunming City, Yunnan Agricultural University; 25°7′N, 102°44′E; 12.VI.2023; 1910 m elev.; coll. Jinghong Li; sweep net (YNAU). ***Paratypes***: China • 1♂; Yunnan, Kunming City, Shimudi ecological park; 25°5′N, 102°50′E; 22.V.2023; 2210 m elev.; coll. Zhizhi Liu; sweep net (YNAU).

#### Diagnosis.

Differs from *S.japonicus* by hindwing media diverging before cu-a; lateral surface of propodeum smooth and shiny anteriorly and medially, and with sparse, oblique, longitudinal rugae posteriorly; pronotal lobe black; mesopleuron with sparse, midsize punctures, episcrobal area finely coriaceous. *Stigmusjaponicus* has the following characters: hindwing media diverging beyond cu-a; lateral surface of propodeum with dense, slender or sturdy, oblique longitudinal rugae anteriorly and medially, and irregular reticulation posteriorly; pronotal lobe ivory; mesopleuron impunctate, episcrobal area with contiguous, longitudinal rugae.

#### Description.

**Female. *Measurements.*** ♀, BL: 5 mm; HW: HLD: HLF = 75: 57: 57; HW: EWd: EW: TW: EL = 75: 18: 22: 28: 52; POD: OOD: OCD = 9: 13: 17; length of scape: length of pedicel: length of flagellomere I: width of flagellomere I: length of flagellomere II: width of flagellomere II = 24: 10: 10: 6: 10: 5; PL: PW: LTI: WTI: HFL: HTL = 38: 10: 40: 40: 41: 40. ♂, BL: 3.8 mm; HW: HLD: HLF = 70: 45: 47: 55; HW: EWd: EW: TW: EL = 70: 22: 22: 20: 46; POD: OOD: OCD = 8: 12: 14; length of scape: length of pedicel: length of flagellomere I: width of flagellomere I: length of flagellomere II: width of flagellomere II = 17: 10: 5: 5: 10: 5; PL: PW: LTI: WTI: HFL: HTL = 35: 10: 34: 25: 40: 49.

***Color pattern.*** Body black; mandible brown except dark brown apically; labrum dark brown; palpi yellowish; scape, pedicel, tegula and forewing veins brown; flagellomeres I–VII brown to dark brown; pronotal lobe black; fore leg: coxa apically, base and apex of femur, tibia largely, tarsi fulvous, remainder brown to dark brown; mid leg: fulvous except middle of trochanter and tibia dark brown; hind leg: trochanter and tibia largely dark brown, remainder fulvous; gaster dark brown apically; clypeal margin and mandible with sparse golden setae.

**Figure 4.**
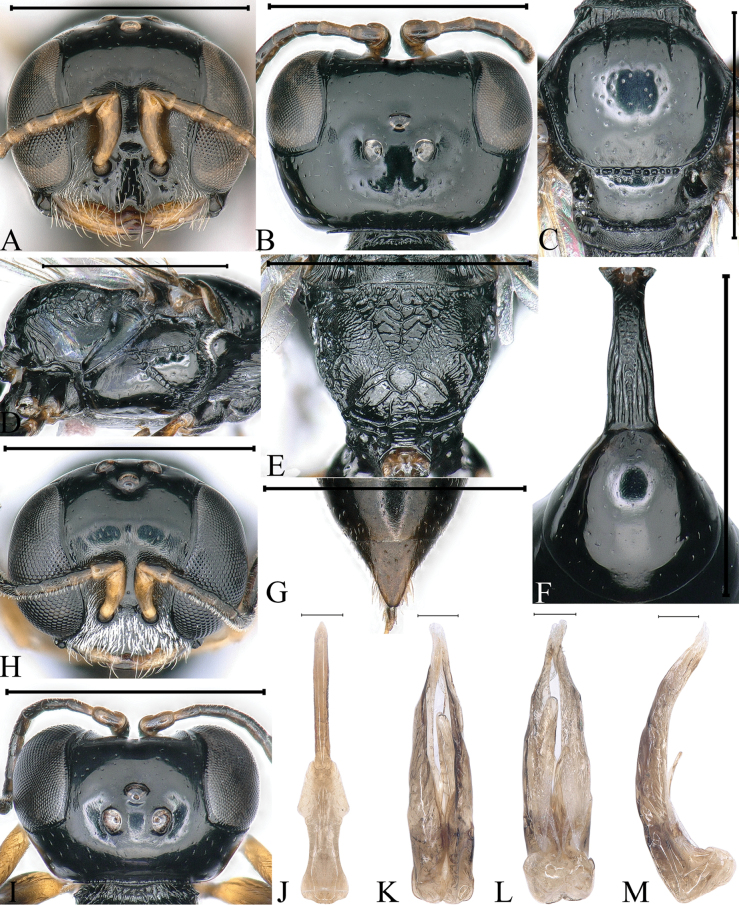
*Stigmusrugidensus* Li & Ma, sp. nov. (**A–G** female **H–M** male) **A, H** head, frontal view **B, I** head, dorsal view **C** collar, scutum, scutellum and metanotum, dorsal view **D** thorax, lateral view **E** propodeum, dorsal view **F** petiole, dorsal view **G** pygidial plate, dorsal view **J** gastral tergum VIII, ventral view **K** male genitalia, dorsal view **L** male genitalia, ventral view **M** male genitalia, lateral view. Scale bars: 1 mm (**A–I**); 0.1 mm (**J–M**).

***Head.*** Mandible tridentate apically, median tooth large. Labrum pentagonal, and with two distinct triangular teeth apically (Fig. 4A). Clypeus shiny, slightly convex, and with sparse, midsize punctures; free margin of clypeus slightly produced and with two distinct, cornuted teeth medially, slightly reflected, distinctly emarginated in middle (Fig. 4A). Scapal hollow coriaceous, somewhat shallow, and clearly defined, provided with one vestigial minute tubercle medially, not spined (Fig. 4A). Frontal furrow very fine and weakly impressed, inconspicuously; median and upper frons shiny, with sparse, midsize punctures mixed with several large punctures, gently convex (Fig. 4A). Ocellar triangle area flat, shiny, impunctate, area near eyes with dense, short, impressed lines, opaque area smaller than hind ocellus (Fig. 4B). Vertex shiny, with sparse, large punctures, and with longitudinal line weakly impressed medially (Fig. 4B). Gena shiny, with sparse, midsize to large punctures dorsally; ventral gena shiny, smooth and impunctate. Head from above with temples rarely convergent posteriorly, subquadrate (Fig. 4B). Occipital carina incomplete, not ending to midventral line, suddenly ended at posterior ridge of stomal hollow, dorsal area narrowed, coarsely crenulate, ventral area slightly broadened, distinctly crenate. Inner and outer orbital furrows lacking (Fig. 4A).

***Mesosoma.*** Anterior area of pronotal collar with strong, transverse carina, and with incomplete lateral rugae, only distinct in posterior area, without antero-lateral corner (Fig. 4C). Scutum shiny, with sparse, large punctures; admedian line distinctly impressed, extending to 2/5 of scutum length; notaulus deeply grooved and crenulate, also reaching 2/5 of scutum length; parapsidal line distinctly impressed (Fig. 4C). Scutellum shiny, with sparse, midsize to large punctures, and without longitudinal line (Fig. 4C). Metanotum slightly matt, finely rugulose (Fig. 4C). Mesopleuron shiny, with sparse, midsize punctures, posterior area smooth, without rugae, episcrobal area finely coriaceous; scrobal suture, omaulus and hypersternaulus broadened, distinctly crenate, scrobal suture complete (Fig. 4D). Propodeal enclosure U-shaped medially, and with a longitudinal median rugae and sparse, irregular, transverse rugae, with dense, disorganized, slender rugae laterally; posterior surface of propodeum with sparse, irregular rugae, median groove inconspicuous (Fig. 4E); lateral surface of propodeum moderately matt, smooth anteriorly and medially, with sparse, oblique longitudinal rugae posteriorly (Fig. 4D).

***Legs.*** Outer surface of hind tibia with three long, slender, fulvous to dark brown spines.

***Wings.*** Forewing venation typical for genus *Stigmus*, hindwing media diverging before cu-a.

***Metasoma.*** Dorsal surface of petiole subquadrate, moderately convex and widened toward apex slightly, and with two sturdy oblique, longitudinal carinae forming V-shaped medially, area between carina with dense, irregular, transverse rugae, median and posterior areas with several sturdy, lateral rugae on each side (Fig. 4F); lateral surface of petiole with two sturdy, longitudinal rugae (Fig. 7E); ventral surface of petiole with a few sturdy, longitudinal rugae posteriorly. Gastral terga shiny, with sparse, midsize punctures, gastral sterna shiny, sterna II–VII with sparse, fine punctures; pygidial area moderately matt, broadly triangular, and with several, midsize punctures basally (Fig. 4G).

**Male.** Same as female, except: mandible fulvous except reddish brown apically; scape and pedicel fulvous; flagellomeres I–III dark brown; fore leg: inner margin of tibia fulvous, middle of femur dark brown; mid leg: trochanter brown, apex of femur and outer margin of tibia dark brown; hind leg: femur, tibia largely black; setae on clypeus dense, silvery, long (Fig. 4H); mandible bidentate apically; clypeus with dense, tiny punctures; free margin of clypeus slightly produced and nearly truncate medially, with shallow emargination, slightly reflected apically (Fig. 4H); vertex shiny, impunctate, without longitudinal line medially (Fig. 4I); head from above with temples somewhat roundly convergent posteriorly (Fig. 4I); flagellomeres without tyloids, setae normal (Fig. 4I); lateral surface of propodeum with irregular reticulation posteriorly (Fig. 7F); gastral sterna moderately matt, impunctate.

#### Distribution.

China (Yunnan).

#### Etymology.

The name, *rugidensus*, is derived from the Latin *rug*- (= rugae) and the Latin word *densus* (= dense), referring to the propodeal enclosure with dense, disorganized, slender rugae on each side.

### ﻿ Stigmus sulciconspicus

Taxon classificationAnimaliaHymenopteraCrabronidae

Li & Ma sp. nov.

10C66C56-758D-5A30-9395-498CE2F9B76C

https://zoobank.org/AFF9DC36-AD65-416E-BDE6-377FF46CE6BD

Figs 5A–G , 8B

#### Type material.

***Holotype***: China • ♀; Baoshan City, Longyang District, Lujiang Country, Gaoligong Mountain; 24°57′N, 98°50′E; 20–21.VII.2006; 938 m elev.; coll. Li Ma; Yellow plate (YNAU). ***Paratypes***: China • 1♀, same data as for holotype.

#### Diagnosis.

Distinguished from *S.interruptus* Bashir & Ma, 2019 by the following combination of characters: anterior area of pronotal collar with complete, sturdy, transverse carina; scutum shiny, with sparse, midsize punctures mixed with a few large punctures, without wrinkle; scrobal suture inconspicuous, just single longitudinal striation; in female, pygidial area shiny, impunctate and without striations. *Stigmusinterruptus* has the following characters: anterior area of pronotal collar with incomplete, sturdy, transverse carina, narrowly emarginated in middle; mesoscutum moderately matt, with sparse, large punctures, anterior and posterior areas with dense, longitudinal micro-striations; scrobal suture broadened, distinctly crenate; in female, pygidial area with weakly longitudinal striations, basal area with several large punctures.

#### Description.

**Female. *Measurements.*** ♀, BL: 4.7 mm; HW: HLD: HLF = 85: 60: 65; HW: EWd: EW: TW: EL = 85: 23: 24: 31: 65; POD: OOD: OCD = 9: 15: 16; length of scape: length of pedicel: length of flagellomere I: width of flagellomere I: length of flagellomere II: width of flagellomere II = 27: 11: 8: 5: 8: 5; PL: PW: LTI: WTI: HFL: HTL = 42: 15: 46: 49: 49: 60.

***Color pattern.*** Body black; clypeus with reddish brown band subapically; mandible yellowish except reddish brown apically; labrum dark brown; palpi yellowish; antenna, tegula, forewing veins and gaster apically fulvous to brown; pronotal lobe white; fore and mid legs: yellowish except coxa largely dark brown; hind leg: coxa largely and femur medially brown, remainder yellowish; clypeal margin and mandible with sparse golden setae.

**Figure 5.**
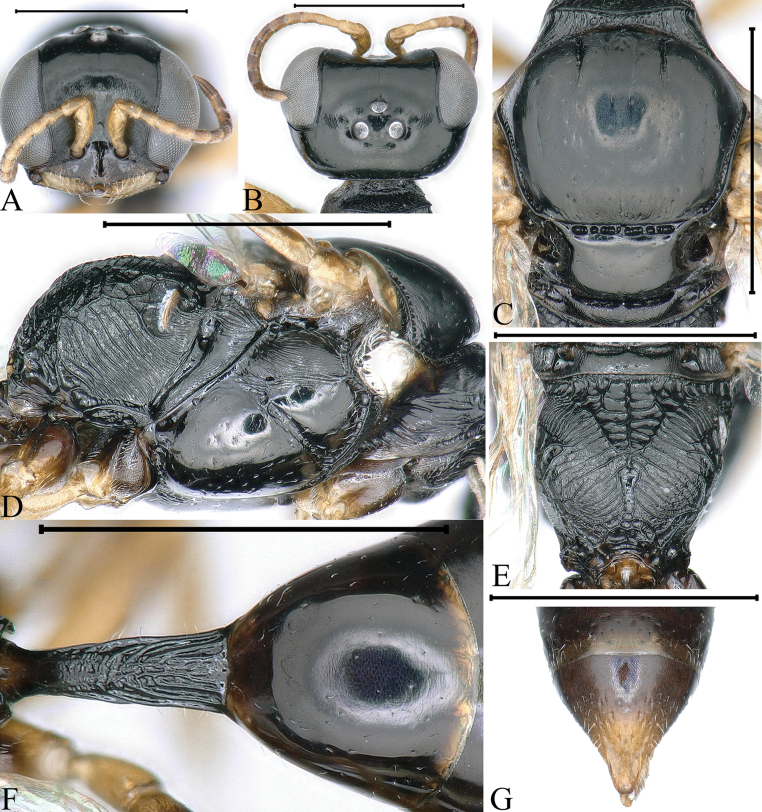
*Stigmussulciconspicus* Li & Ma, sp. nov. (female) **A** head, frontal view **B** head, dorsal view **C** collar, scutum, scutellum and metanotum, dorsal view **D** thorax, lateral view **E** propodeum, dorsal view **F** petiole, dorsal view **G** pygidial plate, dorsal view. Scale bars: 1 mm.

***Head.*** Mandible tridentate apically, median tooth large. Labrum with two distinct, triangular teeth apically (Fig. 5A). Clypeus shiny, moderately convex, and with sparse, fine punctures mixed with several large punctures; free margin of clypeus slightly produced and with two distinct, cornuted teeth medially, slightly reflected, slightly emarginated in middle (Fig. 5A). Scapal hollow shiny, shallow, broad, and not clearly defined, provided with one vestigial, minute tubercle medially, not spined (Fig. 5A). Frontal furrow weakly impressed, inconspicuous (Fig. 5A). Median and upper frons slightly matt, finely coriaceous, and with sparse, midsize punctures, slightly convex (Fig. 5A). Ocellar triangle area flat, shiny, with several fine punctures, area near eyes with dense, short, impressed lines, opaque area larger than hind ocellus (Fig. 5B). Vertex shiny, with sparse, tiny punctures (Fig. 5B). Gena shiny, with sparse, midsize to large punctures dorsally; ventral gena shiny, and with a few midsize punctures. Head from above with temples rarely convergent posteriorly, subquadrate (Fig. 5B). Occipital carina incomplete, not ending to midventral line, suddenly ended at posterior ridge of stomal hollow, without tooth; occipital carina much narrowed, no crenulate. Inner and outer orbital furrows lacking (Fig. 5A).

***Mesosoma.*** Pronotal collar with strong, transverse carina anteriorly, lateral rugae lacking, without antero-lateral corner (Fig. 5C). Scutum shiny, with sparse, midsize punctures mixed with several large punctures; admedian line distinctly impressed, extending to 5/12 of scutum length; notaulus deeply grooved and crenulate, reaching 1/4 of scutum length; parapsidal line distinctly impressed (Fig. 5C). Scutellum shiny, with sparse, tiny punctures, median longitudinal line weakly impressed (Fig. 5C). Metanotum finely coriaceous (Fig. 5C). Mesopleuron shiny, with sparse, midsize punctures, posterior area smooth without rugae, episcrobal area with dense, longitudinal sculptures; scrobal suture inconspicuous, just single longitudinal rugae; hypersternaulus broadened, smooth, not crenate; omaulus broadened, distinctly crenate (Fig. 5D). Propodeal enclosure elongate, U-shaped medially, with one strong, longitudinal, rugae and sparse, irregular, transvers rugae, and with oblique, longitudinal rugae laterally; posterior surface of propodeum with somewhat narrow, shiny, conspicuous, median groove, lateral area of median groove with sparse, oblique, longitudinal rugae, and with sparse, irregular rugae posteriorly (Fig. 5E); lateral surface of propodeum with dense, slender, oblique longitudinal rugae (Fig. 5D).

***Legs.*** Outer surface of hind tibia with three long, slender, fulvous spines.

***Wings.*** Forewing venation typical for genus *Stigmus*, hindwing media diverging beyond cu-a.

***Metasoma.*** Dorsal surface of petiole subquadrate, gently convex and widened toward apex distinctly, with strong, irregular rugae and with several strong, longitudinal rugae posteriorly (Fig. 5F); lateral surface of petiole with a few sturdy, longitudinal rugae (Fig. 8B); ventral surface of petiole with several sturdy, longitudinal rugae posteriorly. Gastral terga shiny, terga I–V with sparse, fine punctures, tergum VI with sparse, midsize punctures apically (Fig. 5G); gastral sterna shiny, sterna I–VI with sparse, fine punctures, sternum VI with dense, fine punctures (Fig. 5G); pygidial area moderately matt, broadly U-shaped, apex rounded (Fig. 5G).

**Male.** Unknown.

#### Distribution.

China (Yunnan).

#### Etymology.

The name, *sulciconspicus*, is derived from the Latin *sulc*- (= groove) and the Latin word *conspicus* (= conspicuous), referring to the posterior surface of propodeum with conspicuous median groove.

### ﻿New record for China

#### ﻿ Stigmus solskyi

Taxon classificationAnimaliaHymenopteraCrabronidae

Morawitz, 1864

70D6636A-6344-579A-9EE5-9523CE33275D

Figs 6A–M , 8C, D

Stigmus solskyi A. Morawitz, 1864: 462; Tsuneki, 1954: 24; Lomholdt, 1975: 129; Bohart & Menke, 1976: 189.
Stigmus europaeus Tsuneki, 1954: 25. Synonymized with Stigmussolskyi by Yarrow, 1954: 239; de Beaumont, 1956: 385; Tsuneki, 1954: 6.
Stigmus verhoeffi Tsuneki, 1954: 6, 26, 36. Synonymized with Stigmussolskyi by de Beaumont, 1956: 385.

##### Specimen examined.

China • 1♀; Inner Mongolia; 8.VII.2001; coll. Bo Qiu. CHINA • 1♀, 3♂♂; Inner Mongolia, Bayan Nur City; 13.VII.2007.

**Figure 6.**
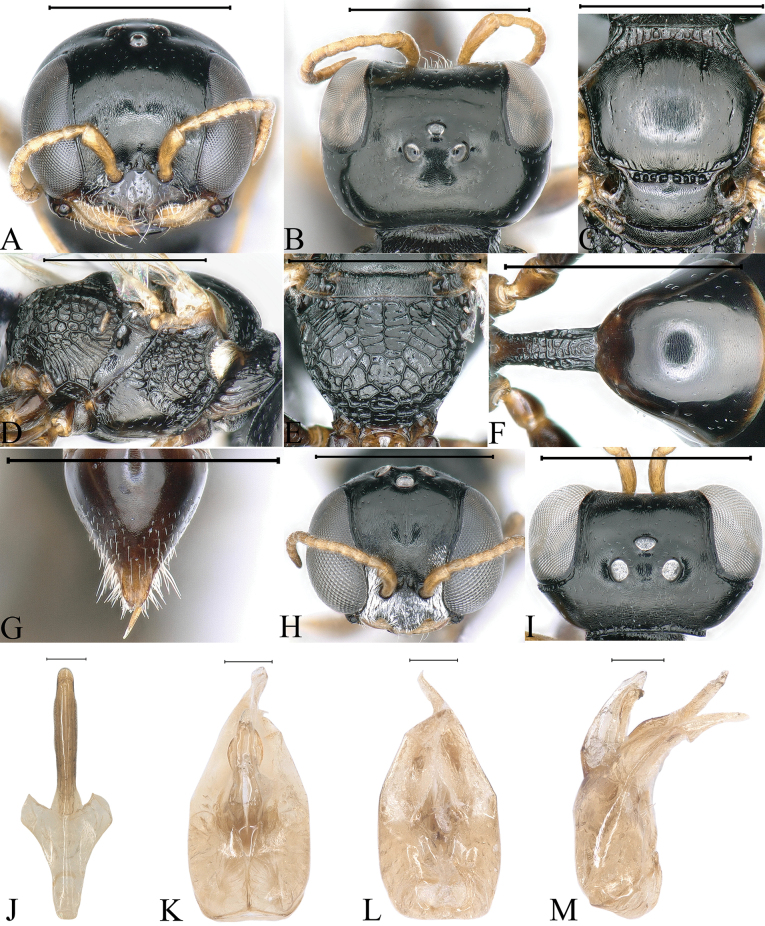
*Stigmussolskyi* Morawitz, 1864. (**A–G** female **H–M** male) **A, H** head, frontal view **B, I** head, dorsal view **C** collar, scutum, scutellum and metanotum, dorsal view **D** thorax, lateral view **E** propodeum, dorsal view **F** petiole, dorsal view **G** pygidial plate, dorsal view **J** gastral tergum VIII, ventral view **K** male genitalia, dorsal view **L** male genitalia, ventral view **M** male genitalia, lateral view. Scale bars: 1 mm (**A–I**); 0.1 mm (**J–M**).

##### Distribution.

China (Inner Mongolia), Algeria, Europe northwards to Finland, Turkey, Georgia, Kazakhstan, Russia.

**Figure 7.**
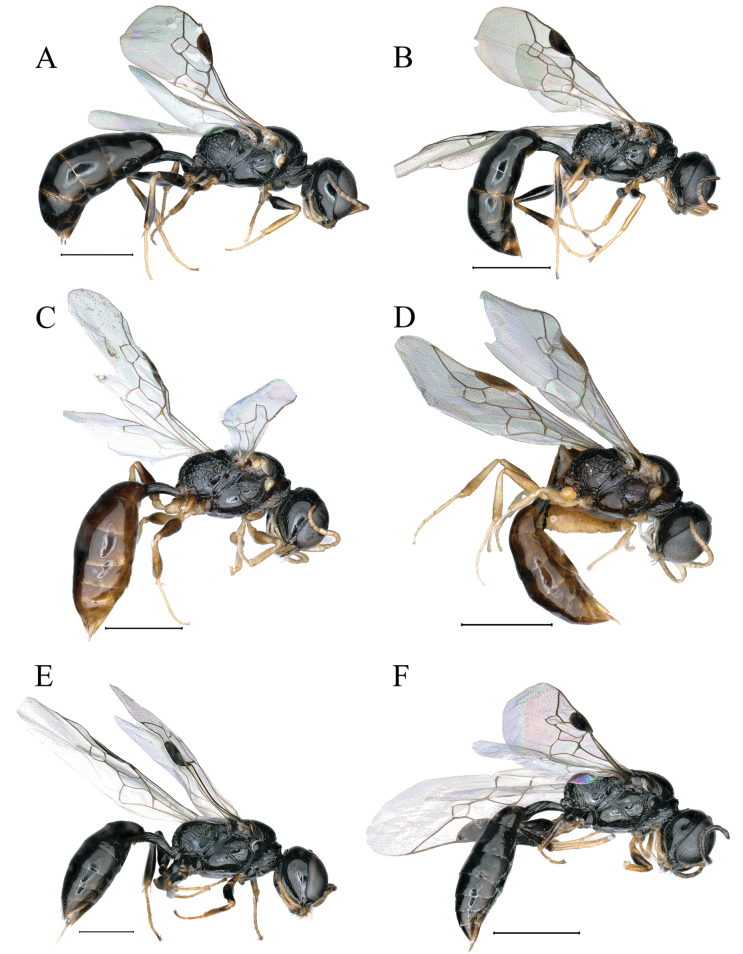
**A, B***Stigmuscarinannulatus* Li & Ma, sp. nov. (**A** female **B** male) **C, D***Stigmusclypeglabratus* Li & Ma, sp. nov. (**C** female, **D** male) **E, F***Stigmusrugidensus* Li & Ma, sp. nov. (**E** female **F** male) **A–F** lateral view. Scale bars: 1 mm.

**Figure 8.**
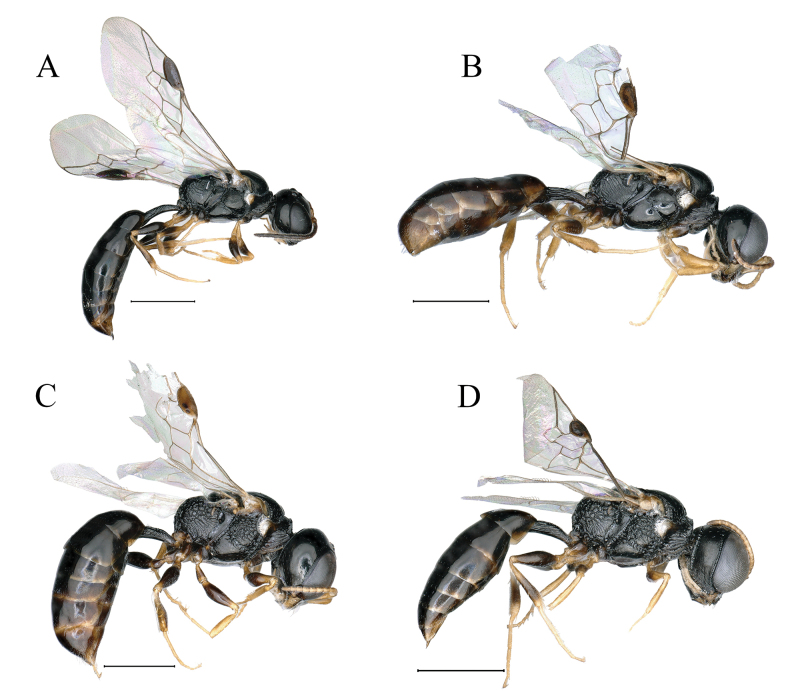
**A***Stigmusflagellipilaris* Li & Ma, sp. nov. (male) **B***Stigmussulciconspicus* Li & Ma, sp. nov. (female) **C, D***Stigmussolskyi* Morawitz, 1864. (**C** female **D** male) **A–D** lateral view. Scale bars: 1 mm.

## Supplementary Material

XML Treatment for Stigmus

XML Treatment for Stigmus carinannulatus

XML Treatment for Stigmus clypeglabratus

XML Treatment for Stigmus flagellipilaris

XML Treatment for Stigmus rugidensus

XML Treatment for Stigmus sulciconspicus

XML Treatment for Stigmus solskyi

## ﻿Additional information

### Author ORCIDs

Jinghong Li https://orcid.org/0009-0002-3346-5018

Qiang Li https://orcid.org/0000-0001-5950-8843

Li Ma https://orcid.org/0000-0002-3436-1387

### Data availability

All of the data that support the findings of this study are available in the main text.
